# Determination of Larval Instars of *Dastarcus helophoroides* (Coleoptera: Bothrideridae) Using Head Capsule Width Frequency Distribution

**DOI:** 10.3390/insects15121013

**Published:** 2024-12-20

**Authors:** Tayyab Shaheen, Jiali Guo, Yun Wang, Jiaojiao Zhou, Guanghui Tang, Zhengqing Zhang

**Affiliations:** 1 Key Laboratory of National Forestry and Grassland Administration on Management of Western Forest Bio Disaster, College of Forestry, Northwest A&F University, Yangling 712100, China; shaheen2339@gmail.com (T.S.); jlguo@nwafu.edu.cn (J.G.); wanyun@nwafu.edu.cn (Y.W.); 15109950399@163.com (J.Z.); 2 Department of Evolutionary Biology and Environmental Studies, University of Zurich, 8057 Zurich, Switzerland

**Keywords:** *Dastarcus helophoroides*, larval instar, frequency distribution, head capsule width

## Abstract

Long-horned beetles are among the major insect pests that can cause significant economic and ecological damage globally. The control of long-horned beetles is crucial to sustain the forest ecosystem. *Dastarcus helophoroides*, an economically important ectoparasitoid of long-horned beetles, is widely utilized in biological control strategies. However, the number of larval instars in *D. helophoroides* remains underexplored. Larval instar determination is crucial for constructing growth prediction models and ecological life tables for insect populations. In this study, we analyzed the frequency distribution of head capsule widths utilizing a visual approach, followed by a non-linear least squares (NLLS) estimation, and found that *D. helophoroides* undergo four larval instars before entering the pupal stage. The theoretical and observed data for each larval instar yielded identical mean Brooks–Dyar’s ratios (1.80). Re-correlation of the number of instars with their respective mean head capsule widths using linear regression (R²) verified that no larval instar was missed. The Crosby’s growth ratio (1% and 2%) indicates a very low likelihood of misclassifying an instar into an adjacent one. Given that the accurate determination of larval instars is crucial for developing effective control programs and predicting future population levels, our findings provide valuable insights for implementing biological control strategies against long-horned beetles.

## Simple Summary

Long-horned beetles can cause severe damage to forest plantations and disrupt forest ecosystems. Controlling their populations is essential for maintaining healthy forests. Biological control, as an efficient and environmentally friendly pest management strategy, can be applied in the early stages of pest development. Laboratory-reared *Dastarcus helophoroides* released into woodlands has proven effective in the sustainable control of long-horned beetle populations. However, the larval instars of *D. helophoroides* have not yet been defined, which complicates field identification. In this study, we measured the head capsule widths of *D. helophoroides* larval instars, visually inspected the histogram, and performed non-linear least squares estimation. Our findings revealed that *D. helophoroides* passes through four larval instars before reaching the pupal stage. Accurately determining the larval instars of a parasitoid is essential for developing effective management programs and predicting future abundances in the field. The findings of this study will be instrumental in implementing biological control strategies for the effective management of long-horned beetles.

## 1. Introduction

*Dastarcus helophoroides* (Fairmaire, 1881) (Coleoptera: Bothrideridae) is a gregarious ectoparasitoid beetle that parasitizes different species of cerambycids, including *Anoplophora glabripennis* (Motschulsky), *Monochamus alternatus* Hope, *Massicus raddei* (Blessig), *Batocera horsfieldi* (Hope), and *Apriona swainsoni* (Hope) [1,2]. Larvae of Cerambycidae can severely impact trees by tunneling into sapwood and even to hardwood as they feed. Affected trees exhibit numerous small tunnels filled with densely packed frass, which compromises their structural stability. Additionally, long-horned beetles of the genus *Monochamus* are known vectors of the pinewood nematode, *Bursaphelenchus xylophilus*, which causes devastating damage to pine trees in East Asia, Europe, and North America [3,4,5]. Therefore, effective management of these long-horned beetles is critical for preserving sustainable ecosystem services. Current control strategies for long-horned beetles predominantly utilize chemical insecticides [6]. However, long-horned beetles spend most of their life stages beneath the bark of the host tree, with the exception of the adult phase; thus, these measures are less effective against larvae. Additionally, chemical insecticides have limited effective duration, pose significant risk to non-target insects, contaminate water, and disrupt ecological balance [7,8,9,10,11]. Introducing *D. helophoroides* eggs or adults into the environment offers a sustainable approach to control long-horned beetles in the field, with successful implementation in China [12,13,14].

Understanding the biology and life cycles of insects is essential for developing the ecological life tables. Furthermore, precise determination of the number of larval stages of an insect without ambiguity is critical for creating accurate growth prediction models [15,16]. Identifying larval instars enables the estimation of developmental durations and alignment of parasitoid life cycles with those of their hosts. This synchronization is crucial to maximize parasitism rates and accurately predict oviposition timing. Larval instars can be identified through a combination of methods, including head capsule measurement (Brooks–Dyar’s rule), microscopic examination, morphological changes, and statistical models [17,18,19]. Brooks–Dyar’s hypothesis estimates the number of larval stages by measuring the width of head capsules across developmental stages [20,21]. This method is based on the principle that the size of the head capsule remains constant within a particular instar but increases geometrically with each successive instar [22]. It has become a foundational approach in studies of instar identification, with a regression model subsequently developed by Gaines and Campbell [23]. Recent studies suggest that kernel density estimation, which analyzes the frequency distribution of head capsule measurements, offers greater accuracy compared to traditional frequency distribution methods [24].

Previous studies have examined the morphology of the adult female reproductive system and identified different external morphological features to distinguish between adult males and females without harming the insects [25,26]. In another study, Xiao et al. [27] investigated the ultrastructure of the antennae and mouthparts of *D. helophoroides* first-instar larvae. However, the total number of larval instars and their mean head capsule widths have not yet been reported, making instar identification in the field challenging. In the present study, the number of larval stages in *D. helophoroides* was identified. Firstly, we measured the head capsule widths of all larval instars and constructed a histogram with fitted curves using Gaussian models. Later, visual inspection of the histogram for larval instar separation at the lowest point between overlapping peaks was followed by nonlinear least-squares (NLLS) parameter estimates to determine *D. helophoroides* larval instars. The findings of this study provide a critical basis for advancing ecological studies and developing effective pest management strategies.

## 2. Materials and Methods

### 2.1. Insect Rearing

The adult specimens of *D. helophoroides* were obtained from the Forest Pest Biological Control Laboratory of the College of Forestry, Northwest Agriculture and Forestry University, Yangling, Shaanxi Province, China. The adults were reared in plastic boxes (dimensions: 5 cm × 5.5 cm × 4.5 cm), nourished with a laboratory-prepared diet composed of crushed silkworm chrysalis powder, egg yolk, sucrose, benzoic acid, and wood flour [28]. Female *D. helophoroides* were permitted to lay eggs on 1.5 × 1.5 cm paper squares attached to cardboard. The eggs were gathered at three-day intervals and deposited in a Petri dish containing a damp cotton pad to ensure adequate moisture levels. *Tenebrio molitor* were reared as hosts for *D. helophoroides* and fed with wheat bran. *T. molitor* offers a good source of proteins, fats, and chitin [29]. Radish was added every two days as a water source. Mature larvae of *T. molitor* were placed inside a 10 mL centrifuge tube [30] with small holes for respiration. Insects were checked for pupation every two days.

Upon eclosion of the first-instar larvae of *D. helophoroides* from eggs, each pupa of *T. molitor* was inoculated with 6 to 7 parasitoid larvae at the abdominal segment utilizing a camel hair brush. Each inoculated *T. molitor* pupae was placed in an autoclaved glass tube (1 cm in diameter and 7.5 cm in length), and the tube opening was sealed with dry cotton [31]. The insects were reared in the breeding room maintained at 24 ± 2 °C with 50 ± 5% relative humidity and an 8:16 h light-to-dark photoperiod.

### 2.2. Head Capsule Measurement

**Figure 1:**
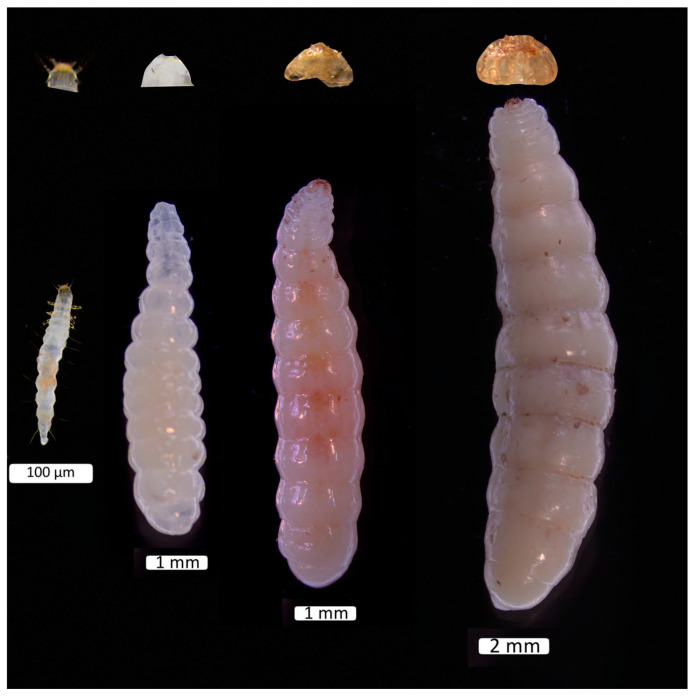
Larval instars of *Dastarcus helophoroides*: 1st, 2nd, 3rd, and 4th (**left** to **right**), along with their excised heads.

To monitor molting, *D. helophoroides* larvae were removed from the host pupa every eight hours. Using a surgical knife under a microscope, the entire head and the first thoracic segment of the larvae were carefully excised. A Wide Zoom Stereo Microscope (SZX16, Olympus, Tokyo, Japan), equipped with an ocular micrometer, was used to measure the head capsule of each larva at 10× magnification, with measurements recorded to the nearest 10 µm. The eyepiece micrometer was calibrated using a stage micrometer (Carter, Zhuzhou, China), and the recorded measurements were converted into actual lengths. Calibration was performed at each magnification setting (ranging from 0.7× to 11.5×: 0.7×, 0.8×, 1×, 1.25×, 1.6×, 2×, 2.5×, 3.2×, 4×, 5×, 6.3×, 8×, 10×, and 11.5×) to ensure maximum precision. The maximum distance between the lateral edges of the larval head capsule was measured to determine its width [32]. Visual representations of *D. helophoroides* across all four instar stages are presented in Figure 1.

### 2.3. Analysis of Head Capsule Widths

The head capsule width data were analyzed by the methods described by Sukovata [33] as “Approach 1”. According to this approach, head capsule measurements were used to create histograms and were visually inspected for larval instar separation at the lowest point between overlapping peaks, followed by NLLS estimation. Frequency distribution analysis, a widely utilized method for determining the number of instars in diverse insect species, proved effective for our study [34,35,36]. The head capsule data of each larval instar, collected from laboratory-reared larvae prior to pupation, were pooled to replicate conditions of those of field collection, resulting in a dataset representing all larval instars without clear demarcation [35]. A histogram of pooled head capsule widths was generated, with instars visually separated at the lowest points between overlapping peaks. Each data subset representing particular instars was used to calculate NLLS and curves fitting a normal distribution to each data subset [33]. The mean and standard deviation of the head sizes in each instar were estimated by the NLLS by fitting a Gaussian curve to each instar peak [37,38]. These estimated parameters served as a foundation for defining the minimum and maximum boundaries of each instar group.

Studies show that the frequency histogram’s peak count aligns with the number of developmental stages in larvae [35,36,37]. Recent studies have shown that frequency distribution analysis incorporating the kernel density estimate function enhances accuracy compared to frequency distribution analysis alone [24,35,36]. Therefore, in this study, we used the seaborn.kdeplot package (version 0.13.2) for analyzing frequency distribution using the kernel density estimate function, where the KDE peaks line up with mean capsule widths. After testing several bandwidth choices, we selected the final values based on optimal goodness-of-fit. Both the theoretical data generated from NLLS analysis and observed head capsule measurements were used for linear regression and second-degree polynomial calculations [23]. Finally, to validate our findings, we calculated Brooks–Dyar’ and Crosby’s growth ratios for each larval instar [22,39,40]. These ratios were obtained using the following equations [41].

Crosby’s index = bn − bn − 1/bn − 1

Brook’s index = Xn/Xn − 1

where bn represents Brook’s index of (n instar) larvae, bn−1 represents Brook’s index of (n − 1 instar) larvae, Xn is the mean measurement for (n instar) larvae, and Xn−1 is the mean measurement for (n − 1 instar) larvae.

All analyses were conducted in Python 3.12 using libraries pandas, matplotlib, NumPy, SciPy, and seaborn.

## 3. Results

### 3.1. Instar-Wise Head Capsule Widths (Observed)

In total, the head capsule width of 293 larvae ranged from 71.41 to 649.93 μm. The head capsule width from the first to fourth larval instars reared on *T. molitor* under laboratory conditions was in the range of 71.41–112.13, 142.83–255.15, 270.18–406, and 466.6–649.93 μm, respectively (Table 1). The observed mean values and standard deviations (SDs) of head capsule width in different instars were 95.52 ± 7.62, 175.64 ± 23.69, 318.01 ± 31.57, and 560.06 ± 43.46, respectively. The Brooks–Dyar’s ratios showed a variation from 1.76 to 1.83, with a mean growth ratio of 1.80 between the first and fourth larval instars. The Brooks-Dyar’s ratio suggested that *D. helophoroides* molted three times and passed through four larval instars before entering the pupal stage. Crosby’s growth ratios showed less than 10% variations among different larval stages (1–2%), showing a reliable larval instar group.

### 3.2. Instar-Wise Head Capsule Widths (Theoretical)

**Figure 2:**
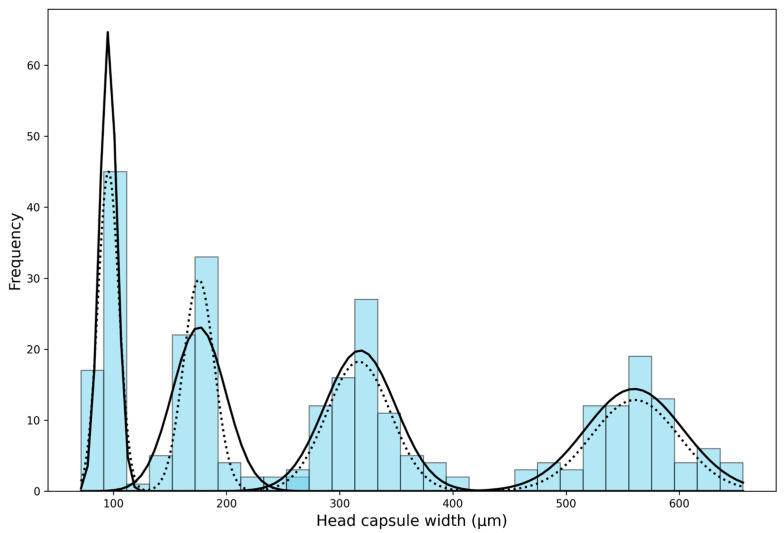
Distribution of measured head capsule widths for *Dastarcus helophoroides*, displayed as a frequency chart. The graph includes fitted curves generated using Gaussian models (represented by a solid black line) and the overall distribution based on non-linear least squares (NLLS) parameter estimates (shown as a black dotted line).

**Figure 3:**
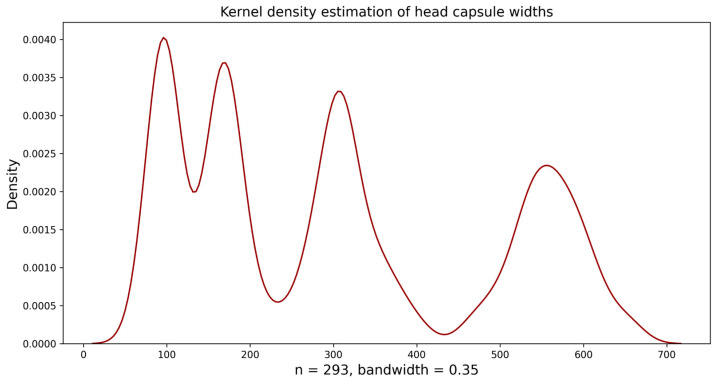
Kernel density estimates (KDEs) of *Dastarcus helophoroides* head capsule widths.

After creating several histograms with varying numbers of bins (intervals), the histogram with 30 bins was visually deemed the most suitable (bin width = 19.49 µm). The histogram revealed four distinct peaks but overlapping in the continuous value range, making the division between the first, second, and third larval instars unclear. The distribution of the fourth instar was non-overlapping. After analyzing the histogram, we subsequently implemented “Approach 1” as outlined by Sukovata [33]; these initial values were used for the normal distribution function (Guassian) to analyze the frequency distribution of each larval instar, wherein curve peaks correspond to the mean of head capsule widths in instars (Figure 2). This mean value was used as a starting value to fit NLLS to the head capsule width data, and values for theoretical means and the standard deviation were calculated. The theoretical means and upper and lower limits differed slightly in the second and third instar. From the first to the fourth instar, the mean head capsule widths were 95.68 ± 9.21, 175.52 ± 13.64, 316.80 ± 28.17, and 560.83 ± 38.78 µm, respectively (Table 2). Individuals in the same instar showed less than 10% variations in their head capsule widths (CV = 4.48–6.30%). The Brooks–Dyar’s ratios between each subsequent instar ranged from 1.77 to 1.83, with an average growth ratio of 1.80; this shows the geometric progression of head capsule width of *D. helophoroides* larval instars. Finally, the Kernel density estimation diagram plotted using *D. helophoroides* head capsule widths showed four distinct peaks at a bandwidth of 0.35, indicating four larval instars of *D. helophoroides* (Figure 3).

### 3.3. Regression Analysis of Head Capsule Widths

**Figure 4:**
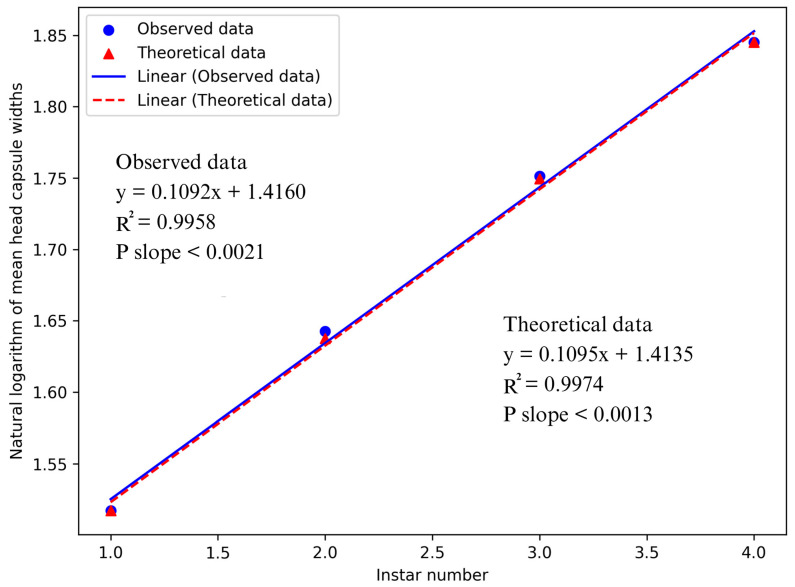
Comparison of observed and theoretical data showing the correlation between *Dastarcus helophoroides* larval instars and their average head capsule widths.

The linear relationship between observed and theoretical data was demonstrated through a regression analysis of head capsule widths and their corresponding instar numbers. This association was evidenced by high R^2^ values of 0.9958 and 0.9974 for the observed and theoretical data, respectively. The linear relationship for both observed and theoretical averages of head capsule widths demonstrated strong statistical significance (*p* < 0.0021 and *p* < 0.0013, respectively). This confirms that *D. helophoroides* goes through four larval instars before reaching its pupal stage and supports Brooks–Dyar’s rule of the geometrical increase in larval head capsules in consecutive instars. The second-degree polynomial regression analysis revealed a linear correlation between instar numbers and mean head capsule widths (R^2^ values of 0.9958 and 0.9974, respectively). As a result, it is proven that no instars were neglected (Figure 4).

## 4. Discussion

We measured the head capsule width of 293 larvae to determine the larval instars of *D. helophoroides*. Our findings indicate that *D. helophoroides* passes through four larval instars before entering the pupal stage. Head capsule width is the most reliable morphological characteristic for distinguishing larval instars in beetles [34]. Consistent with our results, head capsule width has also been identified as a dependable indicator of larval instars in *Dorysthenes granulosus* (Thomson) and *Chilo sacchariphagus indicus* (Kapur) [35,42]. In addition, our findings align with multiple studies that recognize head capsule width as an effective parameter for distinguishing larval instars across various insect species [21,34,36,43]. However, other morphological parameters have been utilized in different studies for larval instar identification [44,45,46,47,48].

In the current investigation, frequency distribution analysis incorporating the kernel density estimate function revealed four distinct peaks that correspond to the four larval instars of *D. helophoroides*. This analysis of larval head capsule widths is commonly used to determine the number of instars in an insect species, with peaks in the histogram typically indicating the distinct instars present [35]. Our findings were consistent with previous studies demonstrating that head capsule width, followed by frequency distribution analysis, serves as a dependable technique to identify instar distribution in various economically significant insect species [24,32,37,44,49,50]. However, Chen and Seybold [24] did not support instar determination through frequency distribution, which may be attributed to the fact that the larvae in their study were not reared individually through all instars until pupation.

Additionally, visual inspection of the histogram combined with NLLS analysis revealed four peaks, representing the four larval instars of *D. helophoroides*. This method was first introduced by McClellan and Logan [37] and further developed by Logan et al. [38]. Consistent with our findings, Delbac et al. [32] and Preto et al. [51] in *Lobesia botrana* and Calvo and Molina [52] and Sukovata [33] in *Streblote panda* and *Dendrolimus pini* also computed NLLS estimates following histogram analysis to determine larval instar. Frequency distribution analysis offers a trustworthy estimate of instar counts [35]. In this work, the number of instars did not deviate from the prediction made by overall frequency distribution analysis based on NLLS and the counts derived from observed data. Similarly, other studies have proved that instar counts observed in the laboratory matched the theoretical numbers derived from frequency distribution analysis of cast head capsule widths [24,35].

In this study, both observed and theoretical data were used to compute Brooks–Dyar’s growth ratios for *D. helophoroides*. Many published studies have employed the Brooks–Dyar’s ratio and regression models to find out the number of instars across various insect species [21,24,38,42,49,53,54]. Ramasubramanian et al. [35] applied the Gaines and Campbell method, finding that head capsule widths accurately identified instars in *C. sacchariphagus indicus*, thereby supporting Brooks–Dyar’s theory. Similar methods were employed to distinguish the instars of *Pissodes castaneus* [20]. According to Brooks–Dyar’s rule for holometabolous insects, the average Brooks–Dyar’s ratio should range from 1.10 to 1.90 [55]. In this research, the average Brooks–Dyar’s ratio for the *D. helophoroides* larval instar was found to be 1.80. However, this ratio exceeds those reported in other studies: 1.46 for *C. sacchariphagus indicus* [35], 1.63 for *Thysanoplusia orichalcea* [56], and 1.51 for *Spodoptera exigua* [24]. In addition, the natural logarithm of mean head capsule widths and the corresponding instars showed a linear relationship. A highly significant linear regression equation for the observed data (*p* < 0.0021; R^2^ = 0.9958) further confirmed a smooth geometric progression in the larval instars of *D. helophoroides*, validating the geometrical development of head capsule widths across successive instars as proposed by Brooks–Dyar’s Law.

Several earlier studies have computed Crosby’s growth ratios to further validate their findings [33,34,36,47,57,58,59]. When the absolute value of Crosby’s growth ratio exceeds 0.1, it suggests that the hypothesized larval instars may be incorrect, and the instar groupings do not conform to the Crosby’s growth rule [36]. Our study showed less than 10% variation among different larval stages (1% and 2%), which validated that our findings are aligned with the Crosby’s growth index. The larval instar number within a species is usually assumed to be constant [60]. Still, numerous factors, including host species, temperature, food quality, photoperiod, humidity, and genetic factors, can affect the number of larval instars [22,61,62]. For example, Guo [63] identified five distinct instars in *A. glabripennis*, while another study reported nine larval instars in the same species. Similar results were observed in *Opisina arenosella* Walker, where larvae reared in the laboratory passed through five to eight instars, while five instars were recorded in the field [64]. In contrast, five larval instars were identified in *M. alternatus* larvae collected from the Sichuan and Zhejiang provinces of China [65,66]. Similarly, the mountain pine beetle, *Dendroctonus ponderosae*, collected from fields in two different Canadian provinces, passed through four larval instars before pupation [67]. In the current study, we report that *D. helophoroides* reared on *T. molitor* pupae under laboratory conditions exhibit four larval instars. However, differences between *D. helophoroides* populations from various hosts and regions should be tested to check their impact on the number of larvae instar.

## 5. Conclusions

*D. helophoroides* is an important parasitoid of *A. glabripennis*, *M. alternatus*, *M. raddei*, *B. horsfieldi*, and *A. swainsoni*. Understanding the life cycle of a parasitoid is crucial for predicting its future populations and developing effective management strategies for its host insects. Since larvae may be more sensitive than adults, understanding instar ratios within the parasitoid larval population can be useful for forecasting population growth and developing control tactics. Therefore, determining the number of larval instar stages is essential for creating ecological life tables. In this investigation, we report that *D. helophoroides* reared on *T. molitor* pupae under laboratory conditions pass through four larval instars before entering the pupal stage.

## Figures and Tables

**Table 1:** Observed mean head capsule widths of larval instars and corresponding Brooks–Dyar’s and Crosby’s growth ratios for *Dastarcus helophoroides*.

Instar	*n*	Head Capsule Width		CV (%)	Brooks–Dyar’s Ratio	Crosby’s Growth Ratio
		Mean ± SD (µm)	Range			
1	63	95.52 ± 7.62	71.41–112.13	4.60		
2	70	175.64 ± 23.69	142.83–255.15	7.78	1.83	
3	80	318.01 ± 31.57	270.18–406	5.73	1.81	−0.01
4	80	560.06 ± 43.46	466.6–649.93	4.48	1.76	−0.02
Mean growth rate					1.80	

*n*: number of larvae; SD: standard deviation; CV: coefficient of variation.

**Table 2:** Theoretical mean head capsule widths of larval instars and corresponding Brooks–Dyar’s and Crosby’s growth ratios for *Dastarcus helophoroides* derived from frequency distribution analysis.

Instar	*n*	Head Capsule Width		CV (%)	Brooks–Dyar’s Ratio
		Mean ± SD (µm)	Range		
1	63	95.68 ± 9.21	˂116.14	4.60	
2	66	175.52 ± 13.64	116.14–235.42	5.37	1.83
3	84	316.80 ± 28.17	235.42–429	6.30	1.80
4	80	560.83 ± 38.78	>429	4.48	1.77
Mean growth rate					1.80

*n*: number of larvae; SD: standard deviation; CV: coefficient of variation.

## Data Availability

The data presented in this study are available on request.
